# Molecular Epidemiology and Genetic Diversity of Norovirus in Young Children in Phnom Penh, Cambodia

**DOI:** 10.1155/2016/2707121

**Published:** 2016-12-27

**Authors:** Kaewkanya Nakjarung, Ladaporn Bodhidatta, Pimmnapar Neesanant, Paphavee Lertsethtakarn, Orntipa Sethabutr, Ket Vansith, Chhour Y. Meng, Brett E. Swierczewski, Carl J. Mason

**Affiliations:** ^1^Department of Enteric Diseases, Armed Forces Research Institute of Medical Sciences, 315/6 Rajvithi Road, Bangkok 10400, Thailand; ^2^National Pediatric Hospital, 100 Russian Federation Boulevard, Phnom Penh, Cambodia

## Abstract

This study investigated the genetic diversity of noroviruses identified from a previous surveillance study conducted at the National Pediatric Hospital in Phnom Penh, Cambodia, from 2004 to 2006. In the previous study, 926 stool samples were collected from children aged 3–60 months with acute diarrhea (cases) and without diarrhea (controls) with reported 6.7% of cases and 3.2% of controls being positive for norovirus. The initial norovirus diagnostic assay was performed with real-time reverse transcription-polymerase chain reaction (real-time RT PCR) which also distinguished between genogroups I and II (GI and GII). Norovirus infection was most commonly detected in children aged 12–23 months in both cases and controls. Norovirus Genotyping Tool and phylogenetic analysis of partial sequences of the 3′ end of the RNA-dependent RNA Polymerase (RdRp) and the capsid domain region were employed to assign genotypes of the norovirus strains. GII.4 was the most predominant capsid genotype detected at 39.5% followed by GII.6 at 14.9%. The GII.4 Hunter 2004 variant was the predominant strain detected. Six RdRP/capsid recombinants including GII.P7/GII.6, GII.P7/GII.14, GII.P7/GII.20, GII.P12/GII.13, GII.P17/GII.16, and GII.P21/GII.3 were also identified. This study of norovirus infection in young children in Cambodia suggests genetic diversity of norovirus as reported worldwide.

## 1. Introduction

Norovirus, a member of the family Caliciviridae, is an important human pathogen and is the leading cause of nonbacterial acute gastroenteritis outbreaks. Norovirus has been increasingly associated with sporadic episodes of acute gastroenteritis in children worldwide. It has been estimated that norovirus infections cause 1 million hospitalizations and 200,000 deaths in children under 5 years of age in the developing world [1].

The norovirus genome is organized into three open reading frames (ORF). ORF1 encodes six nonstructural proteins including the RNA-dependent RNA polymerase (RdRp); ORF2 encodes the capsid; and ORF3 encodes a small, minor structural protein [2]. Noroviruses are classified into at least 6 genogroups (GI-GVI) with a tentative genogroup VII based on the sequence diversity of the RdRP and capsid regions of the genome [3]. Genogroups I, II, and IV are known to infect humans. Genogroups are further subdivided into genotypes and there are 9 GI and 22 GII recognized genotypes based on the capsid sequence [3, 4]. Despite an enormous genetic diversity, the majority of outbreaks and sporadic norovirus cases worldwide are associated with a single genotype from genogroup II, GII.4. Genotype GII.4 was responsible for 62% of reported norovirus outbreaks (4988) in 5 continents from January 2001 to March 2007 [5].

GII.4 variants have been reported as the major cause of norovirus gastroenteritis worldwide starting in 1995 with GII.4 variant Asia 2003 as the most widely circulated variant in Asia during 2003–2006 [6, 7]. In a Peruvian birth cohort study, 97% of characterized repeat norovirus infections were associated with a different genotype or a different GII.4 variant suggesting that genotype-specific immunity may develop with limited cross-protection within the genogroup which highlights the importance of identification and monitoring of GII.4 variants [8].

A potential mechanism that norovirus utilizes to evade host immunity is genetic recombination at the overlapping regions between the RdRp of ORF1 and the capsid protein encoding gene (ORF2), ORF1/ORF2 junction [9]. Multiple recombinants at this region have been reported such as GII.P4/GII.12 and GII.Pb/GII.3 in Japan [10] and GII.P9/GII.4 and GII.P9/GI.7 in Greece [11]. The variability of genetic recombination in norovirus suggests the need for a surveillance system to track the evolution of norovirus. An effective surveillance system would allow a better understanding of the burden of disease caused by norovirus and molecular epidemiology would also facilitate evolutionary analysis of norovirus.

There have been few reports on norovirus variants circulating in Southeast Asia [12–14] and how these norovirus variants compare to variants circulating elsewhere in the world. In the previous study of diarrhea etiology in young children in Phnom Penh, Cambodia, norovirus was the second most common virus detected following rotavirus [12]. In this study, norovirus positive samples from the previous study were further characterized and norovirus molecular epidemiology is reported including GII.4 variants and norovirus recombinants.

## 2. Materials and Methods

### 2.1. Study Design

A detailed description of the study design has been reported previously [12]. Briefly, children aged 3 months to 5 years were enrolled at the National Pediatric Hospital (NPH) in Phnom Penh between November 2004 and October 2006. Cases were enrolled among inpatient and outpatient children with acute diarrhea of no more than 72 hours' duration. Controls were children who visited the same hospital for other reasons and had not had diarrhea in the previous two weeks. Informed consent was obtained from one parent or a guardian for each participant. The study was approved by institutional review boards in both Cambodia and the United States.

### 2.2. Stool Collection and Nucleic Acid Extraction

Approximately 3–5 g of stool was collected from subjects. Stool samples were stored at −70°C until processed. A 10% (wt/vol) stool suspension was prepared with distilled sterile water and total nucleic acids were extracted with NucliSens® Magnetic Extraction Kit (BioMerieux Inc., Durham, NC, USA) following the instructions of the manufacturer.

### 2.3. Real-Time Reverse Transcription (RT) PCR Screening and Genogrouping for Norovirus

The extracted nucleic acids were screened to identify the genogroup (GI and GII) by real-time RT PCR reactions as described previously [15]. The reactions were set up using the TaqMan® EZ RT PCR Core Reagent kit (Applied Biosystems, Foster City, CA, USA). All reactions were carried out in ABI PRISM 7900 Sequence Detector System and the results were analyzed with Sequence Detection Software version 2.1 (Applied Biosystems, Foster City, CA, USA).

### 2.4. Reverse Transcription (RT) PCR for the Cloning of ORF1/ORF2 Junction Regions

The extracted nucleic acids of norovirus positive samples were treated with DNase (Invitrogen, Carlsbad, CA, USA) to remove DNA prior to reverse transcription reaction. Five *μ*L of RNA was reverse transcribed with primer G1SKR for GI and G2SKR for GII [16] to generate cDNA of the ORF1/ORF2 junction region using Multiscribe® Reverse Transcriptase (Invitrogen, Carlsbad, CA, USA).

The ORF1/ORF2 junction was amplified using AmpliTaq Gold® polymerase (Applied Biosystems, Foster City, CA, USA) containing a mixture of three forward primers (G1FF (A, B, and C) for GI or G2FB (A, B, and C) for GII) and reverse primer (G1SKR for GI or G2SKR for GII) [16, 17]. The thermocycling profile used was heat activation at 95°C for 10 min, 40 cycles of denaturation at 95°C for 30 sec, annealing at 48°C for 30 sec, extension at 72°C for 1 min, and postincubation at 72°C for 7 min.

PCR products (GI 597 bp and GII 468 bp) were cloned into a TA-Cloning vector (pCR 4.0-TOPO, Invitrogen, Carlsbad, CA, USA). One to three positive clones were sequenced from both forward and reverse directions using a commercial sequencing service (Macrogen, Seoul, Korea). DNA sequencing data were verified for consensus sequence using Sequencher software version 4.1.2 (Gene Codes Corporation, Ann Arbor, MI, USA).

### 2.5. Phylogenetic Analysis

The identification of norovirus genotype was performed by submitting sequences of the junction between RdRP and capsid genes to the online Norovirus Genotyping Tool (Version 1.0) [4]. Phylogenetic trees were also constructed based on sequences of RdRP and capsid genes. Nucleotide sequences of GI (448 bp correspond to nucleotides 4929 to 5376 of U07611 strain) and GII (436 bp corresponds to nucleotides 4929 to 5366 of U07611 strain) were aligned with representative reference strains (GI.Pa/GI.3-GQ856473, GI.Pc/GI.5-AB039774, GI.Pd/GI.3-GQ856470, GI.P8/GI.8-GU299761, GII.P2/GII.2-X81879, GII.P3/GII.3-AB112332, JN176920, GII.P6/GII.6-JX989075, GII.P7/GII.7-AF414409, GII.P7/GII.6-AB504694, KM198549, GII.P7/GII.14-EF670650, GII.P7/GII.20-AB542917, GII.P12/GII.12-AB525813, GII.P12/GII.13-AB354294, GII.P13/GII.13-EU921354, GII.14-AY130761, GII.P16/GII.16-GQ856476, GII.P16/GII.17-AY502009, GII.P17/GII.17-KC597139, GII.P20/GII.20-EU275779, GII.P21/GII.21-EU019230, GII.P21/GII.3-KM198586, and GIII.1-EU360814 (out group)). For GII.4 variants determination, the nucleotide sequences from GenBank were used: GII.4 Yerseke 2006a-AB447433, GII.P12/GII.4 Asia 2003-AB220922, and GII.4 Hunter 2004-HM802542. Sequences of norovirus partial RdRp and capsids genes were submitted to GenBank.

All sequences were aligned with ClustalW [18] in MEGA Version 6 [19]. Phylogenetic tree was constructed in MEGA Version 6 using neighbor-joining with Kimura two-parameter model with 1,000 bootstrap replicates.

### 2.6. Recombination Analysis

Simplot version 3.5.1 was used to identify putative recombination breakpoints by comparing query sequence to nonrecombinant and recombinant parental strains [20]. Analysis parameters were as follows: Window: 200 bp, Step: 20 bp, GapStrip: On, Kimura (2-parameter), and T/t: 2.0.

### 2.7. Statistical Analysis

The differences among proportions were analyzed by chi-square test and the difference between means was analyzed by* t*-test in IBM® SPSS® Statistics Version 22 (IBM Corp., Armonk, NY, USA).

## 3. Results

### 3.1. Norovirus Detection

**Figure 1:**
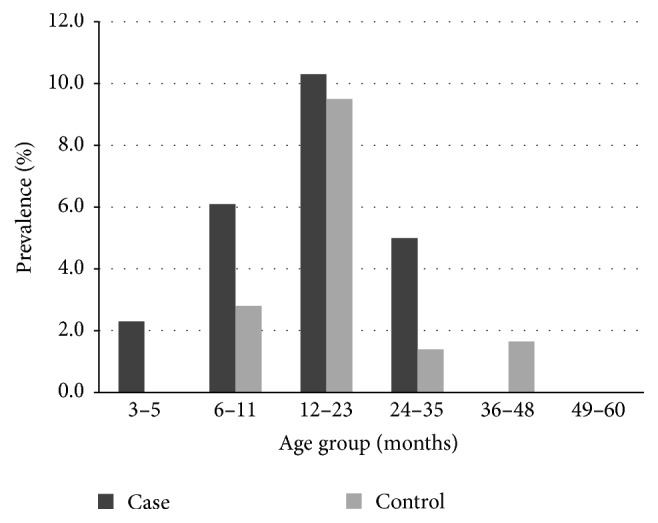
Prevalence of norovirus GI and GII infection in children with acute diarrhea (cases) and nondiarrheal controls by age. Samples were collected from children aged 3 months to 5 years who attended the National Pediatric Hospital in Phnom Penh, Cambodia, between November 2004 and October 2006. A significant difference (*p* < 0.05,* t*-test) in the mean of age of infection was observed between cases and controls averaging at 12.4 months and 20.3 months, respectively.

A total of 926 stool samples (580 cases and 346 controls) were previously tested for the presence of norovirus and other enteric pathogens [12]. The prevalence of GI in cases and controls was 0.7% and 0.9% and the prevalence of GII was 6.0% and 2.3%, respectively (Table 1) [12]. Children in the age group of 12–23 months had the highest prevalence in both cases and controls at 10.3% and 9.5%, respectively (Figure 1). There is a significant difference in the mean ages of cases and controls with a mean age and standard deviation of 12.4 ± 5.3 months and 20.3 ± 10.58 months, respectively (Figure 1).

### 3.2. Sequence and Phylogenetic Analysis

Amplification of the ORF1/ORF2 junction region was successfully performed on 39 (7 cases and 32 controls) out of the 50 real-time PCR norovirus positive samples (2 GI and 37 GII). Repeated attempts to amplify the remaining 11 positive samples were unsuccessful. Identification of norovirus genotypes was achieved by cloning PCR products and performing sequence analysis on positive clones. The cloned PCR product corresponded to a 597 (GI) and a 468 (GII) bp fragment that maps to the overlapping region of ORF1 and ORF2.

**Figure 2:**
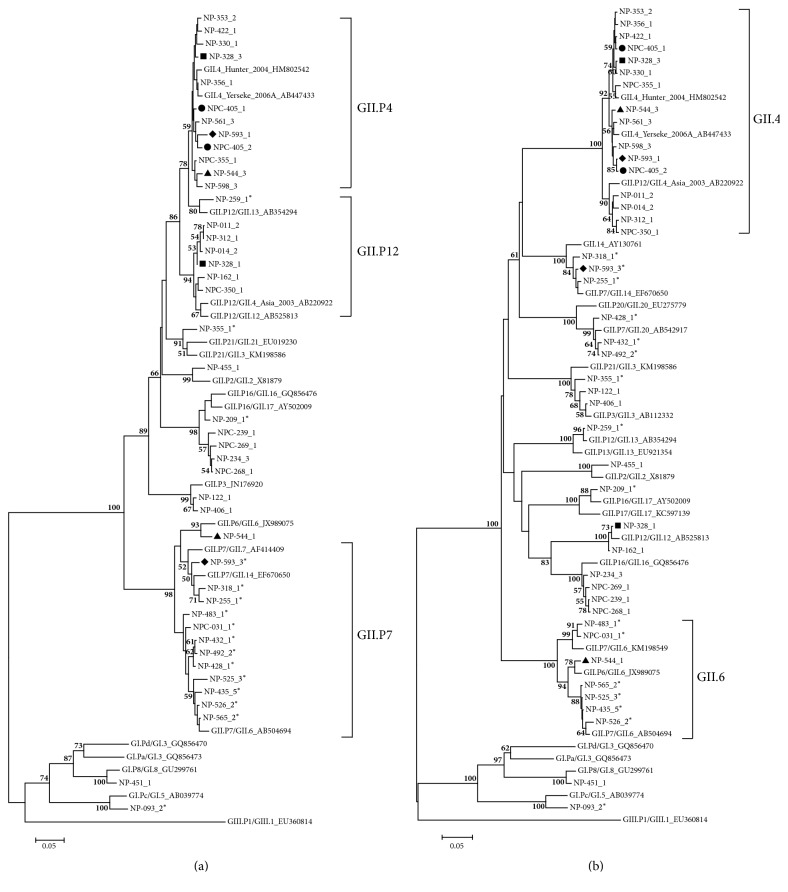
Phylogenic analysis of partial RNA-dependent RNA polymerase (RdRP) (a) and capsid genes (b) of norovirus from Cambodia pediatric samples during 2004–2006. Sample names are indicated as NP ###-# for case and NPC ###-# for control. -# after the sample name indicates colony number of clone for that sample. Recombination strains are indicated by ^*∗*^  at the end of sample name. Mixed infections from same sample are indicated with similar symbol (■ for NP-328, ● for NPC-405, ▲ for NP-544, and ♦ for NP-593).

A total of 43 nucleotide sequences were obtained. Four additional sequences were of mixed norovirus infection from the same sample. GenBank accession numbers of all sequences are KX685457–KX685499. These sequences were then submitted to the online Norovirus Genotyping Tool to identify their genotypes based on partial RdRP and capsid genes. GII.4 was the most predominant capsid genotypes assigned at 39.5% followed by GII.6 at 14.9% (Table 2). GII.4 capsid variants that were identified included GII.4 Asia 2003, GII.4 Hunter 2004, and GII.4 Yerseke 2006a; however, 3 GII.4 sequences were not assigned any variant by the Norovirus Genotyping Tool. RdRP genotypes were assigned to 51.2% (22/43) of nucleotide sequences. Phylogenetic analysis of RdRP sequences clustered unassigned sequences into GII.P3 (1), GII.P4 (12), GII.P7 (5), GII.P12 (2), and GII.P21 (1) (Figures 2(a) and 2(b) and Table 2).

Two or more consensus genotype sequences were derived from 4 samples suggesting a mixed infection in these samples (Figures 2(a) and 2(b)). Mixed infections in this study are both inter- and intragenotype which included GII.P12/GII.12 with GII.P4/GII.4 Hunter 2004, GII.P6/GII.6 with GII.P4/GII.4 Yerseke 2006a, GII.P4/GII.4 Asia 2003 with GII.P7/GII.14, and GII.P4/GI.4 Hunter 2004 with GII.P4/GII.4 Asia 2003.

### 3.3. Recombination Analysis

Both of the Norovirus Genotyping Tool and phylogenetic analysis revealed 7 different recombinant genotypes from 16 sequences. GII.P7/GII.6 was the predominant recombinant with 6 sequences followed by GII.P7/GII.14 and GII.P7/GII.20 at 3 sequences each. Other recombinants were GI.Pc/GI.5, GII.P12/GII.13, GII.P16/GII.17, and GII.P21/GII.3 at 1 sequence each (Table 2). GII.P7 was the predominant RdRP that recombine with other capsid genotypes (GII.6, GII.14, and GII.20); however, there is no statistical significance between GII.P7 recombinants in case versus control samples in comparison to other recombinants identified in the study.

**Figure 3:**
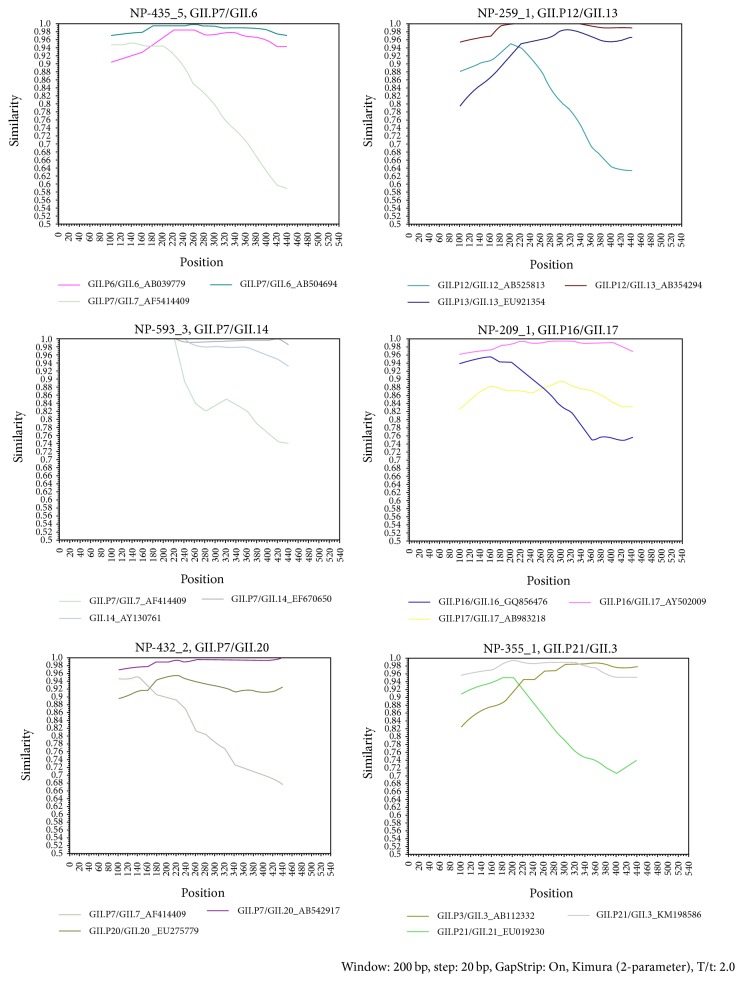
Representative plots of Simplot analysis of each norovirus recombinant. The *y*-axis indicates a degree of similarity between query recombinant and recombinant and two nonrecombinant parental norovirus strains. Sample name and recombination genotype are located on top of each plot. Samples names are indicated as NP ###-# for case and NPC ###-# for control. # after the sample name indicates colony number of clone for that sample. Nonrecombinant parental strains used are GII.P3/GII.3-AB112332, GII.P6/GII.6-AB039779, GII.P7/GII.7-AF414409, GII.P12/GII.12-AB525813, GII.P13/GII.13-EU921354, GII.14-AY130761, GII.P16/GII.16-GQ856476, GII.P17/GII,17-AB983218, GII.P20/GII.20-EU275779, and GII.P21/GII.21-EU019230. Recombinant parental strains used are GII.P7/GII.6-AB504694, GII.P7/GII.14_EF670650, GII.P7/GII.P20-AB542917, GII.P12/GII.13-AB354294, GII.P16/GII.17-AY502009, and GII.P21/GII.3-KM198586.

All of the 16 recombinant sequences were subjected to Simplot analysis to determine recombination breakpoints. Table 2 shows a range of nucleotide breakpoint of each recombinant in comparison to the reference strain Lordsdale, accession number X86557, which falls into ORF1/ORF junction. Representatives of Simplot of each recombinant are shown in Figure 3.

## 4. Discussion

The presence of norovirus in cases of pediatric diarrhea in Cambodia was described previously but little is known about the genetic diversity of the circulating norovirus strains [12, 29]. In this study, the percentage of norovirus positive cases among children with diarrhea seen at the hospital (6.7%) is relatively low compared to studies from neighboring Southeast Asian countries [13, 14, 30]. This does not necessarily reflect the true burden of norovirus gastroenteritis in Cambodia for several reasons. One of the possible limiting factors was that it was a passive surveillance where sample collection was performed at a single hospital where possible sample bias can be introduced. Additionally, the low percentage is perhaps from underreported norovirus diarrhea cases to the tertiary care hospital as norovirus associated diarrhea may not be severe or it is an uncommon practice to seek healthcare for diarrheal disease in Cambodia. The finding from a community-based surveillance study in Cambodia reported that, even among severe cases of diarrhea, less than 30% sought treatment from a healthcare facility [31]. Additionally, it may be possible that norovirus is overshadowed by the presence of other pathogens in low-income settings where sanitary measures are limited as evidenced by higher percentages of bacteria and rotavirus detected in the previous report [12, 32]. Norovirus prevalence has become more prominent in higher-income settings where other pathogens are controlled through improvement of public health control measures for water and sanitation [32]. Additional systematic surveillance will be required to fully understand the burden of norovirus infection in Cambodia.

Age distribution of norovirus infection in this study was similar to what has been reported elsewhere [33–35]. Children in the 3–5-month age group were not commonly infected with norovirus, possibly due to maternal immunity and the protective benefit of breast-feeding [36]. The prevalence was highest in both cases and controls in the 12–23-month age range and declined after 24 months of age. Information on age distribution of norovirus infection is important for targeting population for norovirus vaccine implementation.

Studies of sequence and genotypic analysis of norovirus positive samples described GII.4 as the most common genotype detected in both outbreaks and sporadic cases of acute gastroenteritis worldwide [5]. The 16 isolates of GII.4 identified in this study were classified into 3 variants: Hunter 2004, Yerseke 2006a, and Asia 2003, corresponding to reported norovirus genotypes circulating during 2004–2006 worldwide [7, 37, 38]. Mixed norovirus infections were also detected in 4 cases which might serve as a potential source of inter- and intragenotype norovirus recombination.

Recombination between the RNA-dependent RNA polymerase and the capsid genes has been reported worldwide in recent years [39]. The Norovirus Genotyping Tool and phylogenetic tree analysis of the Cambodian norovirus sequences led to the identification of 7 recombinants with more than 95% sequence identity to published sequences of recombinant variants (Figure 3 and Table 2). Simplot program was used to confirm 5 recombination genotypes and identified recombination breakpoints at the ORF1/2 junction which correspond to previously reported breakpoints (4981–5117 nucleotides of Lordsdale virus genome) [27, 40]. Recombination breakpoint could not be confirmed for GI.Pc/GI.5 and GII.P7/GII.14 as nonrecombinant parental strains are not available [3].

All of the recombinants identified in this study have been reported previously from various geographical locations from samples that were collected prior to, after, or at the same time period as this study. GI.Pc/GI.5 and GII.P21/GII.3 were reported earlier than the rest of recombinants in this study with GII.P21/GII.3 being the most widely detected recombinant [9, 21, 41]. The rest of recombinants identified in this study match recombinants reported in 2008 or later suggesting that recombinants identified in this study existed before. However, due to geographical distances, it is likely that norovirus recombinants identified in this study occurred as a separate event and may not be directly related to reported recombinants. Approximately 37% of norovirus isolates (16/43) in this study were recombinants which suggest that viral recombination has an important role in norovirus success in an evasion of host immune responses as supported by an increase in reports of novel recombinant strains [9, 39].

Despite advances in understanding norovirus biology, no norovirus virulence determinants have been identified and there is currently no efficient way to predict which strains will become dominant. Sequence and biochemical studies suggest that norovirus employs at least two mechanisms to persist in the population: antigenic drift and recombination [7, 42]. Understanding how norovirus evolves and adapts to immunological pressure is critical for the development of an effective vaccine and antiviral therapy.

## 5. Conclusions

This study reports on molecular epidemiology of norovirus circulating in young children in Phnom Penh, Cambodia, form 2004 to 2006. The existence of several GII.4 variants and recombinant strains in Cambodia suggests the need for a continued surveillance system that includes molecular aspects to provide a better epidemiological understanding for the development of vaccines against norovirus.

## Figures and Tables

**Table 1:** Norovirus infection classified by genogroups in stool samples from children (3 months to 5 years) with acute diarrhea (cases) and nondiarrheal controls who attended the National Pediatric Hospital in Phnom Penh, Cambodia, between November 2004 and October 2006.

	Cases (*N* = 580)	Controls (*N* = 346)
Norovirus	39 (6.7%)^*∗*#^	11 (3.2%)^*∗*#^
GI	4 (0.7%)	3 (0.9%)
GII	35 (6.0%)^*∗*^	8 (2.3%)^*∗*^

^**∗**^ *p* < 0.05, chi-square test.

^#^Meng et al., 2011.

**(a):** 

Genotypes		Number of samples	
ORF1	ORF2	Case	Control
GI.P8	GI.8	1	0
GII.P2	GII.2	1	0
GII.P3	GII.3	2	0
GII.P4	GII.4 Asia 2003	2	1
GII.P12	GII.4 Asia 2003	3	1
GII.P4	GII.4 Hunter 2004	5	2
GII.P4	GII.4 Yerseke 2006a	3	0
GII.P6	GII.6	1	0
GII.P12	GII.12	2	0
GII.P16	GII.16	1	3

**(b):** 

Recombinants						
Genotypes		Number of samples		Recombination position	Lordsdale nucleotide position	First report
ORF1	ORF2	Case	Control			
GI.Pc	GI.5	1	0	—	—	Japan 1997–2001 [21]
GII.P7	GII.6	5	1	101–128	5022–5049	Burkina Faso 2011 [22]
GII.P7	GII.14	3	0	—	—	China [23]^#^
GII.P7	GII.20	3	0	97–109^*∗*^	5022-5034	Brazil 2008 [24]
GII.P12	GII.13	1	0	144–152	5065–5073	South Korea 2004–2007 [25]
GII.P16	GII.17	1	0	191–201	5112–5122	South Africa 2010–2012 [26]
GII.P21	GII.3	1	0	147–156	5067–5076	Europe, Australia, Japan, 2002-2003 [10, 27, 28]

Recombination breakpoint positions of each genotype and corresponding positions in the reference strain Lordsdale are reported.

^*∗*^Numbering based on NP-492_2.

^#^The original publication described the recombinant as GII.P6/GII.14 (GenBank accession number EF670650) but the Norovirus Genotyping Tool and phylogenetic analysis showed that it was closely related to GII.P7/GII.14.
